# Polo-like kinase 1 (PLK1) and protein phosphatase 6 (PP6) regulate DNA-dependent protein kinase catalytic subunit (DNA-PKcs) phosphorylation in mitosis

**DOI:** 10.1042/BSR20140051

**Published:** 2014-06-25

**Authors:** Pauline Douglas, Ruiqiong Ye, Laura Trinkle-Mulcahy, Jessica A. Neal, Veerle De Wever, Nick A. Morrice, Katheryn Meek, Susan P. Lees-Miller

**Affiliations:** *Departments of Biochemistry & Molecular Biology and Oncology, Southern Alberta Cancer Research Institute, University of Calgary, 3330 Hospital Drive NW, Calgary, AB, Canada, T2N 4N1; †Department of Cellular & Molecular Medicine and Ottawa Institute of Systems Biology, University of Ottawa, Ottawa, Ontario, Canada, K1H 8M5; ‡Departments of Pathobiology & Diagnostic Investigation, and Microbiology & Molecular Genetics, Michigan State University, East Lansing, Michigan, U.S.A.; §Department of Biological Sciences, University of Calgary, 2500 University Drive NW, Calgary, AB, Canada T2N 4N1; ∥Beatson Institute for Cancer Research, Glasgow G61 1BD Scotland, U.K.

**Keywords:** DNA-dependent protein kinase, midbody, mitosis, polo-like protein kinase 1, protein phosphatase 6, ATM, ataxia telangiectasia mutated, Chk2, checkpoint kinase 2, DMEM, Dulbecco’s modified Eagle’s medium, DNA-PKcs, DNA-dependent protein kinase catalytic subunit, DAPI, 4′,6-diamidino-2-phenylindole, DSB, DNA double-strand break, FHA, forkhead associated, GFP, green fluorescent protein, IR, ionizing radiation, MEM, Minimum Essential Medium Alpha, NHEJ, non-homologous end-joining, PIPES, 1,4-piperazinediethanesulfonic acid, PLK1, polo-like kinase-1, PP6, protein phosphatase 6, siRNA, small interfering RNA, TPX2, targeting protein for Xklp2

## Abstract

The protein kinase activity of the DNA-PKcs (DNA-dependent protein kinase catalytic subunit) and its autophosphorylation are critical for DBS (DNA double-strand break) repair via NHEJ (non-homologous end-joining). Recent studies have shown that depletion or inactivation of DNA-PKcs kinase activity also results in mitotic defects. DNA-PKcs is autophosphorylated on Ser^2056^, Thr^2647^ and Thr^2609^ in mitosis and phosphorylated DNA-PKcs localize to centrosomes, mitotic spindles and the midbody. DNA-PKcs also interacts with PP6 (protein phosphatase 6), and PP6 has been shown to dephosphorylate Aurora A kinase in mitosis. Here we report that DNA-PKcs is phosphorylated on Ser^3205^ and Thr^3950^ in mitosis. Phosphorylation of Thr^3950^ is DNA-PK-dependent, whereas phosphorylation of Ser^3205^ requires PLK1 (polo-like kinase 1). Moreover, PLK1 phosphorylates DNA-PKcs on Ser^3205^ *in vitro* and interacts with DNA-PKcs in mitosis. In addition, PP6 dephosphorylates DNA-PKcs at Ser^3205^ in mitosis and after IR (ionizing radiation). DNA-PKcs also phosphorylates Chk2 on Thr^68^ in mitosis and both phosphorylation of Chk2 and autophosphorylation of DNA-PKcs in mitosis occur in the apparent absence of Ku and DNA damage. Our findings provide mechanistic insight into the roles of DNA-PKcs and PP6 in mitosis and suggest that DNA-PKcs’ role in mitosis may be mechanistically distinct from its well-established role in NHEJ.

## Abstract 2

PLK1 (polo-like kinase 1) phosphorylates and PP6 (protein phosphatase 6) dephosphorylates DNA-PKcs (DNA-dependent protein kinase catalytic subunit) in mitosis. DNA-PKcs autophosphorylation and DNA-PKcs dependent phosphorylation of Chk2 in mitosis occur in the absence of Ku and DNA damage.

## INTRODUCTION

The DNA-PKcs (DNA-dependent protein kinase catalytic subunit) plays critical roles in NHEJ (non-homologous end-joining), the major pathway for the repair of IR (ionizing radiation)-induced DSBs (DNA double-strand breaks) in human cells [1–3]. NHEJ is also required for V(D)J recombination and thus functional T and B cells in the vertebrate immune system [4,5]. Moreover, merging evidence suggests that NHEJ is also required for the prevention of chromosomal translocations and deletions, thus preventing genomic instability, an enabling hallmark of cancer [6]. The protein kinase activity of DNA-PKcs is required for successful completion of NHEJ and V(D)J recombination [7,8] and DNA-PKcs autophosphorylates on multiple sites *in vitro* [9]. Many *in vitro* DNA-PKcs autophosphorylation sites, including Ser^2056^, Thr^2609^, Thr^2638^, Thr^2647^ and Thr^3950^ are phosphorylated in a DNA-PK-dependent manner after DNA damage indicating that DNA-PKcs undergoes autophosphorylation *in vivo* [10–12]. Thr^2609^, Thr^2647^ and Thr^2638^ can also be phosphorylated by the related protein kinases ATM (ataxia telangiectasia mutated) and ATR (ATM- and Rad3-related) [13,14], whereas Ser^3205^ is phosphorylated exclusively by ATM after DNA damage [15]. Cells expressing DNA-PKcs in which the Thr^2609^ cluster of phosphorylation sites (also referred to as the ABCDE cluster) has been mutated to alanine are extremely radiation sensitive, have DSB repair defects and, in V(D)J recombination, have coding joint defects, consistent with decreased end processing [10,11,16]. In contrast, DNA-PKcs with mutation at the Ser^2056^ cluster of autophosphorylation sites (also called the PQR cluster) has increased end processing at coding joints [17] indicating that autophosphorylation of DNA-PKcs at different sites can have reciprocal effects on DNA-PKcs function (reviewed in [10]). Substitution of DNA-PKcs-dependent *in vivo* phosphorylation site Thr^3950^ (located in the putative activation loop of DNA-PKcs) with the phosphomimic aspartic acid abrogated DNA-PK enzymatic activity and NHEJ, suggesting that autophosphorylation at this site negatively regulates DNA-PKcs protein kinase activity [18]. In contrast, *in vitro* autophosphorylation site Ser^3205^ is phosphorylated in an ATM-dependent manner after DNA damage; however, ablation of Ser^3205^ did not induce radiation sensitivity [15] and its function remains unknown.

Given the importance of DNA-PK autophosphorylation in NHEJ, we searched for protein phosphatases that might regulate DNA-PKcs protein kinase activity and function. DNA-PKcs interacts with PP6 (protein phosphatase 6), which is composed of catalytic (PP6c) and regulatory subunits (including SAPS1, 2 and 3–also known as PP6R1, PP6R2 and PP6R3, respectively) [19,20]. PP6 was shown to be required for activation of DNA-PKcs [20]; however, PP6 did not dephosphorylate DNA-PKcs at Ser^2056^ or Thr^2609^ after IR [19,20], and how PP6 regulates DNA-PKcs function is uncertain. Recently PP6 was shown to dephosphorylate the mitotic protein kinase Aurora A on the regulatory Thr^288^ in the kinase activation loop, inhibiting its activity [21]. Moreover, siRNA (small interfering RNA) depletion of PP6c interfered with mitotic spindle formation and chromosome alignment due to increased Aurora A protein kinase activity [21,22], revealing the important roles for PP6 in mitosis.

Recent studies have also uncovered new roles for DNA-PKcs in mitosis. Proteomics studies have identified DNA-PKcs at mitotic spindles [23–26] and depletion of DNA-PKcs or inhibition of its protein kinase activity leads to misalignment of mitotic chromosomes as well as other features of abnormal mitoses [23,27]. Moreover, DNA-PKcs is phosphorylated on Ser^2056^, Thr^2609^ and Thr^2647^ in mitosis, and phosphorylation at these sites is DNA-PK-dependent [23,27]. DNA-PKcs phosphorylated on Thr^2609^ and Thr^2647^ localizes to centrosomes and DNA-PKcs phosphorylated on Thr^2609^ is found at kinetochores in metaphase and at the midbody as cells approach cytokinesis [23,27]. Thus, in addition to its well-established role in DSB repair, emerging evidence reveals unexpected roles for DNA-PKcs in mitosis.

Here, we show that DNA-PKcs is also phosphorylated on Thr^3950^ and Ser^3205^ in mitosis. Like phosphorylation of Ser^2056^ and Thr^2609^, mitotic phosphorylation of Thr^3950^ is DNA-PK-dependent and DNA-PKcs phosphorylated at Thr^3950^ localizes to the midbody in cytokinesis. In contrast, phosphorylation of DNA-PKcs on Ser^3205^ in mitosis requires PLK1 (polo-like kinase 1). Moreover, DNA-PKcs interacts with PP6 in mitosis and PP6 dephosphorylates Ser^3205^ when cells exit mitosis. Although DNA-PKcs phosphorylates Chk2 protein kinase on Thr^68^ in mitosis [28,29] to regulate BRCA1 Ser^988^ phosphorylation [29,30], inhibition of PLK1 did not affect Chk2 phosphorylation at Thr^68^, suggesting that PLK1-dependent phosphorylation of DNA-PKcs and DNA-PKcs-dependent phosphorylation of Chk2 represent independent pathways in mitosis. Moreover, autophosphorylation of DNA-PKcs and DNA-PKcs-dependent phosphorylation of Chk2 in mitosis appear to be independent of both Ku and DNA damage. Collectively our data provide mechanistic insight into the role of DNA-PKcs and PP6 in mitosis and suggest that DNA-PKcs’ role in mitosis is mechanistically distinct from its well-established roles in NHEJ and V(D)J recombination.

## MATERIALS AND METHODS

### Reagents and antibodies

Microcystin-LR, BSA, PMSF, Tris base, EGTA, leupeptin and pepstatin were purchased from Sigma-Aldrich. The ATM inhibitor (KU55933) was from Tocris. The PLK1 inhibitor (BI2536), the DNA-PK inhibitor (NU7441) and the Aurora A inhibitor (Aurora A Inhibitor-1) were purchased from Selleck Chemicals. Antibodies to PP6c, SAPS1, SAPS2 and SAPS3 were from Bethyl Laboratories. Antibodies to DNA-PKcs and CEP55 were from Abcam. Phosphospecific antibodies to Ser^3205^ and Thr^3950^ of DNA-PKcs and antibodies to total DNA-PKcs were as in [15,18]. Antibodies to actin, α -tubulin and FITC-conjugated α-tubulin were from Sigma-Aldrich. Antibodies to TPX2 (targeting protein for Xklp2) and Chk2 total were from Novus, whereas the phosphospecific antibody to phospho-Thr^68^ of Chk2 was from Cell Signalling. Antibodies to cyclin B and lamin A/C were from Santa Cruz. The phosphospecific antibody to phospho-Thr^210^ of PLK1 was from BD Pharmingen. The antibody to Ku 70 was as in [31]. Antibodies to total PLK1, Aurora A and Aurora A phospho-Thr^288^ were purchased from Abcam, Serotec and Cell Signalling, respectively.

### Cell culture

HeLa cells were cultured in DMEM (Dulbecco's modified Eagle's medium) (Invitrogen) supplemented with 5% (v/v) FBS (Hyclone), 50 units/ml penicillin and 50 μg/ml streptomycin. U2OS cells were cultured in the RPMI 1640 medium (Invitrogen) supplemented with 10% (v/v) FBS (Hyclone), 50 units/ml penicillin, and 50 μg/ml streptomycin. Ku null xrs6 rodent cells were cultured in the MEM (Minimum Essential Medium Alpha) (Invitrogen) supplemented with 10% (v/v) FBS (Hyclone), 50 units/ml penicillin, 50 μg/ml streptomycin and 2.5 μg/ml blasticidin. Ku null xrs6 rodent cells stably expressing GFP (green fluorescent protein)-tagged human DNA-PKcs were cultured in MEM (Invitrogen) supplemented with 10% (v/v) FBS (Hyclone), 50 units/ml penicillin, 50 μg/ml streptomycin and 0.4 μg/ml G418 as described [32]. DNA-PKcs null rodent cells (V3), stably expressing human wild-type DNA-PKcs were cultured in DMEM (Invitrogen) supplemented with 10% (v/v) FBS (Hyclone), 50 units/ml penicillin, 50 μg/ml streptomycin and 0.4 mg/ml G418 as described previously [15]. All cells were maintained at 37°C under a humidified atmosphere of 5% (v/v) CO_2_.

### Preparation of cell extracts from asynchronous cells

Asynchronously growing HeLa cells were harvested and detergent (NP-40) lysates were prepared as in [19]. Where indicated, cells were irradiated 10 Gy using a ^137^Cs source as described previously [19], allowed to recover for 1 h or as indicated and harvested as above.

### Immunoblotting and immunoprecipitation

Immunoblotting and immunoprecipitation were carried out as in [19]. Where indicated, the broad-spectrum nuclease benzonase (Sigma Aldrich) was added to cell extracts (5 units/mg total protein) prior to the preclear step in immunoprecipitation experiments to disrupt protein–DNA and protein–RNA mediated interactions.

### Isolation of mitotic spindles

Mitotic spindle preparations were isolated from taxol and nocodazole-treated cells according to the published procedures, resulting in a preparation enriched for mitotic spindles as well as kinetochores and centrosomes [24,33]. Where indicated, extracts were prepared in the presence of protein phosphatase inhibitors (1 μM microcystin-LR, 50 mM NaF and 10 mM sodium orthovanadate) to preserve phosphorylation-dependent interactions [34]. Briefly, HeLa cells were grown to mid-confluency, and incubated with thymidine (2 mM) for 17 h. Thymidine-containing medium was removed, cells were washed in the fresh medium and after 7 h placed into the media containing nocodazole (40 ng/ml). After a further 7 h, cells were harvested by mitotic shake off and left to recover in fresh media for 35 min until most of them reached metaphase [monitored by immunofluorescence analysis of DAPI (4′,6-diamidino-2-phenylindole)-stained cells]. Microtubules were subsequently stabilized with paclitaxel at 5 μg/ml for 3 min at 37°C. Cells were then harvested, washed with PBS containing 2 μg/ml latrunculin B, 1 mM PMSF, 5 μg/ml taxol, and then incubated for 15 min at 37°C in lysis buffer: 100 mM PIPES (1,4-piperazinediethanesulfonic acid) (pH 6.9), 1 mM MgSO_4_, 2 mM EGTA, 0.5% (v/v) Nonidet P40, 5 μg/ml taxol, 2 μg/ml latrunculin B, including nucleases (200 μg/ml DNase I, 10 μg/ml RNase A, 1 unit/ml micrococcal nuclease, 20 units/ml benzonase), protease inhibitors (1 μg/ml pepstatin,1 μg/ml leupeptin, 1 μg/ml aprotinin, 1 mM PMSF) and, where indicated, protein phosphatase inhibitors (1 μM microcystin-LR, 50 mM NaF and 10 mM sodium orthovanadate). Lysed cells were centrifuged at 700×***g*** for 2 min and resuspended in the same buffer, incubated for 5 min and harvested again by centrifugation. The supernatant (Fraction 1) was boiled in SDS sample buffer. Mitotic spindles were isolated by incubating the lysed cells in isolation buffer [1 mM PIPES–KOH (pH 6.9), 1 mM PMSF, 5 μg/ml paclitaxel] for a further 10 min and collected by centrifugation at 1500×***g*** for 3 min. The supernatant (Fraction 2) was removed and mixed with SDS cocktail. The remaining pellet was resuspended in [25 mM Tris–HCl (pH 7.5), 0.1 mM EGTA, 0.1% β-mercaptoethanol, 1 mM benzamidine, 0.1 mM PMSF] with 600 mM NaCl and incubated for 10 min at room temperature before centrifugation at 52000×***g*** for 35 min. The supernatant was diluted to 420 mM salt (Fraction 3) and resuspended in SDS sample buffer.

### Preparation of extracts from mitotic cells

Where indicated, cells were incubated with nocodazole (40 ng/ml) for 16 h, then harvested by shake off and lysed either immediately, or placed in fresh, nocodazole-free media and harvested after the recovery times indicated in the figure legends. Detergent extracts were generated as described above. To confirm that nocodazole treatment induced mitosis, an aliquot of cells was harvested after the nocodazole treatment and after shake off, stained for histone H3–Ser^10^ phosphorylation and analysed by flow cytometry as described previously [19]. At least 60% of the nocodazole-treated cells in the shake off fraction stained positive for H3-phospho-Ser^10^, confirming that they were enriched for mitotic cells (Supplementary Figure S1 at http://www.bioscirep.org/bsr/034/bsr034e113add.htm).

### siRNA transfection

SMARTpool siRNA oligonucleotides were purchased from Dharmacon (Lafayette). Transfection was carried out as described in [19]. Twenty-four hours after transfection, fresh medium was added. At 80 h cells were either left untreated or treated with 40 ng/ml nocodazole for 16 h, then harvested by mitotic shake-off and analysed as described in the figure legends. Alternatively, 24 h after transfection, cells were irradiated with 10 Gy and harvested 1 h post-recovery as described above.

### Immunofluorescence

Cells were grown on poly-L-lysine-coated coverslips and treated with the indicated protein kinase inhibitors as described in the figure legends. Cells were fixed and analysed as described in [19].

### *In vitro* phosphorylation of DNA-PK by PLK1

His tagged PLK1 purified from baculovirus-infected insect cells was a kind gift from Dr James Hastie and Dr Dario Alessi, University of Dundee, Scotland, U.K. DNA-PKcs and Ku were purified from HeLa cells as described previously [35]. Purified DNA-PK (equimolar amounts of DNA-PKcs and Ku) was incubated with His-tagged purified PLK1, in the absence or presence of 5 μM of the DNA-PK inhibitor (NU7441) or 100 nM of the PLK1 inhibitor (BI2536) in 25 mM Hepes-NaOH pH 7.5, 50 mM KCl, 10 mM MgCl_2_, 1 mM dithiothreitol, 0.2 mM EGTA, 0.1 mM EDTA plus 10 μg/ml sonicated calf thymus DNA as indicated. Reactions were started by the addition of 0.25 mM ATP and reactions were incubated at 30°C for 30 min then stopped by addition of SDS sample buffer and analysed on SDS–PAGE followed by Western blotting as above.

## RESULTS

### DNA-PKcs, Ku and PP6 localize to mitotic spindles

**Figure 1:**
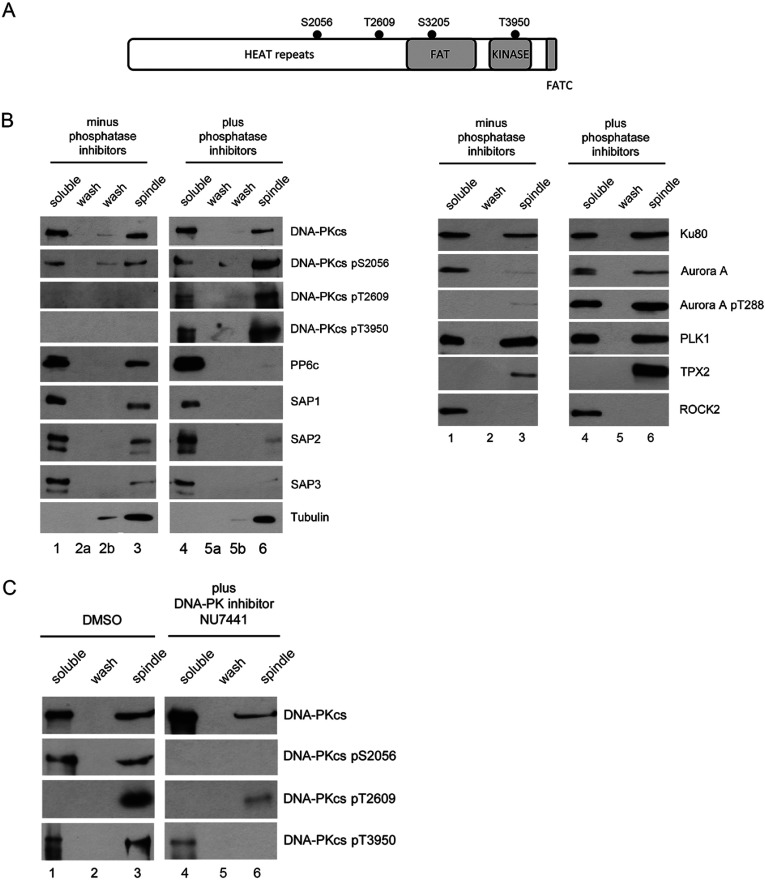
DNA-PKcs binds to and is phosphorylated at enriched mitotic spindles (**A**) Schematic representation of DNA-PKcs showing major domains and positions of Ser^2056^, Thr^2609^, Ser^3205^ and Thr^3950^ phosphorylation sites. (**B**) Mitotic spindles and associated proteins were isolated according to [33] either in the absence (left) or presence (right) of protein phosphatase inhibitors (see the Materials and Methods section for details). Lanes 1 and 4 contained soluble proteins. Lanes 2 and 5 contained the low ionic strength wash, and lanes 3 and 6 the mitotic spindle fraction (see the Materials and Methods section for details). In the blots shown in the left-hand panel, an additional low salt wash (lanes 2b and 5b) was included on the gels. Samples were boiled in SDS–PAGE sample buffer, run on SDS–PAGE gels, transferred to nitrocellulose and probed with the described antibodies. (**C**) Mitotic spindles were prepared as in Figure 1(B) with or without the addition of the DNA-PK inhibitor (NU7441) 1 h prior to mitotic shake off. Samples were run on SDS–PAGE, transferred to nitrocellulose and probed for phosphorylation as indicated.

DNA-PKcs has a well-established role in NHEJ and is phosphorylated on multiple sites including Ser^2056^, Thr^2609^, Ser^3205^ and Thr^3950^ in response to DNA damage (Figure 1A). Recent studies have also revealed a novel role for DNA-PKcs in mitosis. siRNA depletion of DNA-PKcs or inactivation of DNA-PKcs with the small molecule inhibitor NU7441 led to a significant increase in misaligned mitotic chromosomes as well as an increase in the number of lagging chromosomes in mitosis ([23,27] and Supplementary Figure S2 at http://www.bioscirep.org/bsr/034/bsr034e113add.htm).

To explore further the roles of DNA-PKcs and PP6 in mitosis, HeLa cells were synchronized by thymidine/nocodazole block and extracts were fractionated to enrich for mitotic spindles. Where indicated, extracts were prepared in the presence of protein phosphatase inhibitors to preserve serine/threonine phosphorylation events and phosphorylation-dependent interactions [34]. Fractions were analysed by SDS–PAGE, and probed for DNA-PKcs, PP6 and known mitotic proteins. DNA-PKcs was detected in spindle fractions and, when extracts were prepared in the presence of protein phosphatase inhibitors, DNA-PKcs was phosphorylated on Ser^2056^, Thr^2609^ and Thr^3950^ (Figure 1B). Also present in the spindle fraction were Ku80, and, as expected, mitotic proteins, Aurora A, polo-like kinase 1 (PLK1) and TPX2 (Figure 1B). Spindle preparations were also probed for the cytoplasmic protein kinase ROCK2 as a negative control. PP6c and its regulatory subunits SAPS1, SAPS2 and SAPS3 were also enriched in the mitotic spindle fraction when extracts were prepared in the absence of protein phosphatase inhibitors, suggesting that either the protein phosphatase activity of PP6 is required for its association with spindles, or that phosphorylation of a spindle component is required to anchor PP6 at mitotic spindles (Figure 1B).

### Mitotic phosphorylation of DNA-PKcs on Thr^3950^ is DNA-PK-dependent and Thr^3950^ phosphorylated DNA-PKcs localizes to centrosomes in prometaphase and the midbody at cytokinesis

Given that phosphorylation of DNA-PKcs at Ser^2056^, Thr^2609^ and Thr^2647^ in mitosis is DNA-PK-dependent [23,27], we next asked whether phosphorylation of Thr^3950^ was also DNA-PK-dependent. Mitotic spindles were prepared as above but treated with the DNA-PKcs inhibitor NU7441 for 1 h prior to harvest. As shown in Figure 1(C), phosphorylation on Thr^3950^ was also DNA-PK-dependent in mitosis.

**Figure 2:**
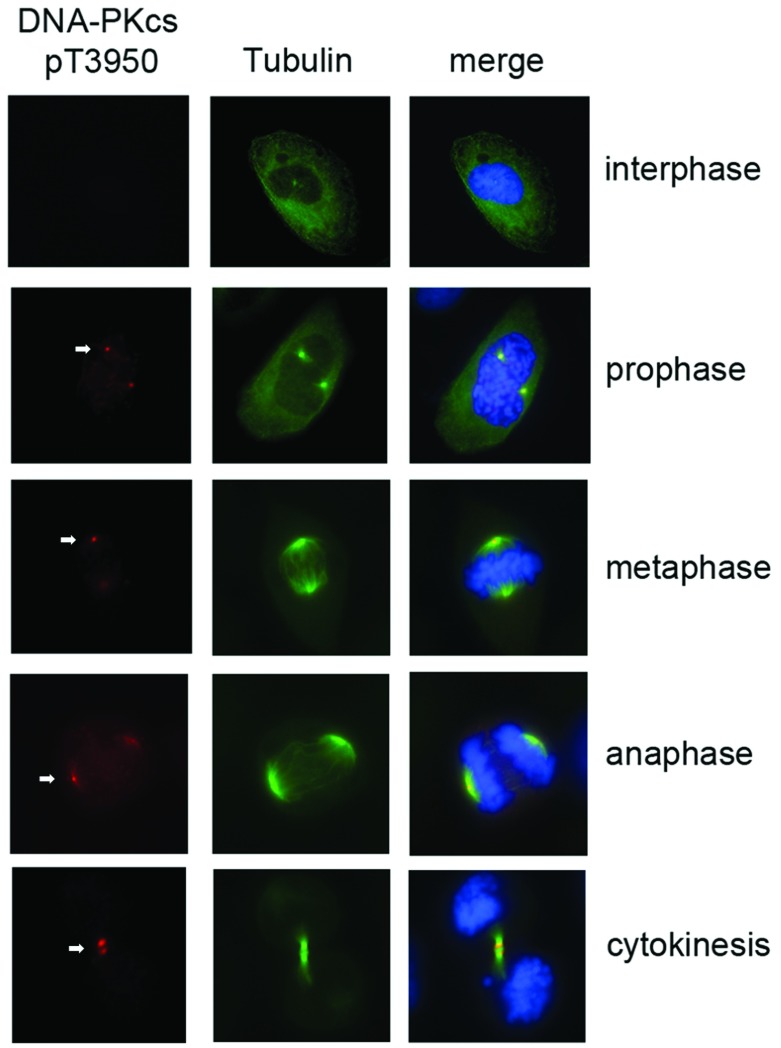
Phosphorylation of DNA-PKcs at Thr^3950^ in mitotic cells U2OS cells were stained with FITC-conjugated α-tubulin (green) at 1:1000 dilution, and phosphospecific antibody to DNA-PKcs phospho-Thr^3950^ (red) at 1:200 dilution at different phases of the cell cycle. DNA stained with DAPI is shown in blue. An expanded image of a cell in cytokinesis stained with the DNA-PKcs phosphoThr^3950^ antibody (panel B) is shown in Supplementary Figure S3(A).

DNA-PKcs phosphorylated on Ser^2056^, Thr^2609^ and Thr^2647^ localizes to centrosomes in metaphase, and DNA-PKcs phosphorylated on Thr^2609^ localizes to the midbody in cytokinesis [23,27]. To further explore the mechanism by which DNA-PKcs functions in mitosis, the subcellular localization of DNA-PKcs phosphorylated on Thr^3950^ was determined by immunofluorescence using a phosphospecific antibody to DNA-PKcs phospho-Thr^3950^. Preliminary experiments were carried out on HeLa cells; however, U2OS osteosarcoma cells were used for subsequent experiments as the percentage of mitotic cells that remained attached to the coverslips was found to be higher in U2OS cells than in HeLa cells. Thr^3950^-phosphorylated DNA-PKcs was absent in interphase U2OS cells but was detected at centrosomes during prophase and metaphase (Figure 2A). Like phosphosphorylation at Thr^2609^, DNA-PKcs phosphorylation at Thr^3950^ was detected at the midbody at cytokinesis (Figure 2 and Supplementary S3A at http://www.bioscirep.org/bsr/034/bsr034e113add.htm), as was Thr^210^ phosphorylated PLK1 (Supplementary Figure S3B).

### PLK1 phosphorylates DNA-PKcs at Ser^3205^ in mitosis

**Figure 3:**
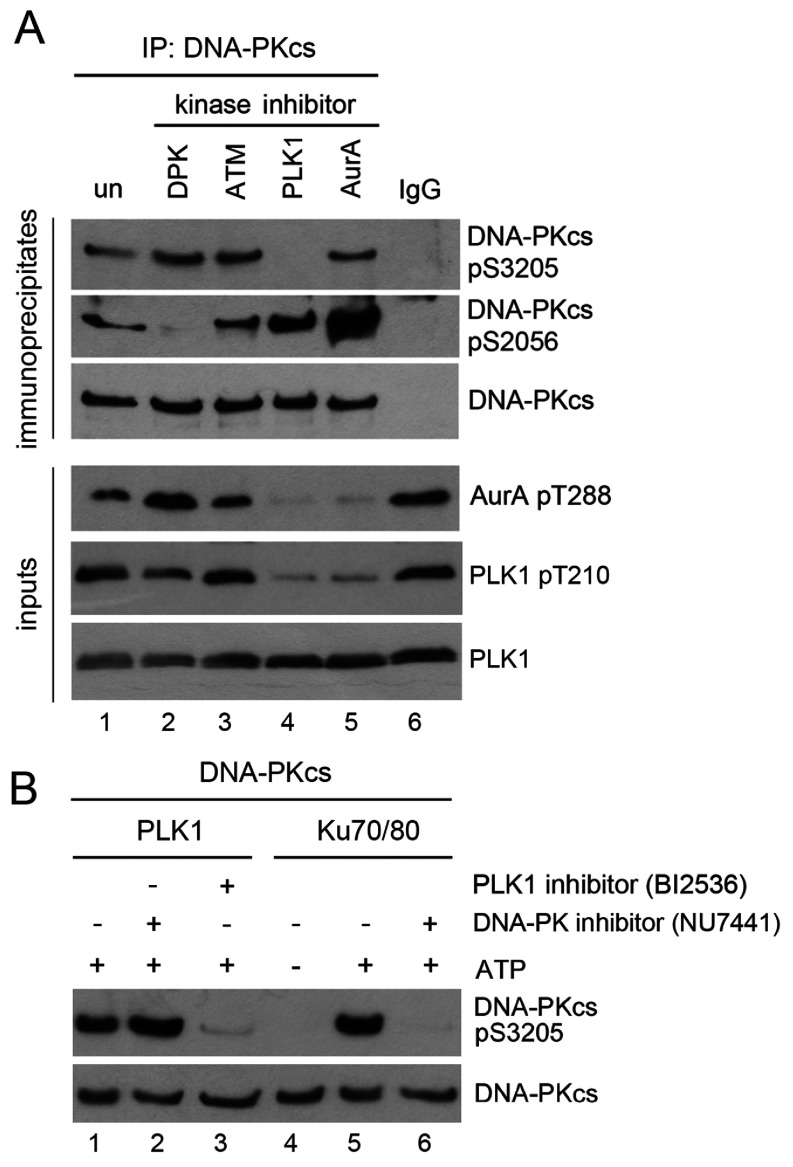
PLK1 phosphorylates DNA-PKcs on Ser^3205^ in mitosis (**A**) HeLa cells were treated with 40 ng/ml nocodazole for 15 h, then 1 h prior to shake off either mock-treated (un) or treated with 8 μM of the DNA-PK inhibitor (NU7441), 5 μM of the ATM inhibitor (KU55933), 100 nM of the PLK1 inhibitor (BI2536) or 100 nM Aurora-A-Inhibitor-I for 1 h. After shake off, cells were allowed to recover in fresh media, in the absence of nocodazole but in the continued presence of kinase inhibitors, for a further 35 min before harvesting by centrifugation. NETN lysis was carried out and DNA-PKcs was immunoprecipitated from 1 mg of lysate as described in the Materials and Methods section. Immunoprecipitates were blotted with the described antibodies. The lower panel shows 50 μg of the whole cell extract probed for Aurora A Thr^288^ and PLK Thr^210^ phosphorylation as well as total PLK1 to show efficacy of PLK1 and Aurora A inhibitors. (**B**) Purified DNA-PKcs was incubated with His-tagged purified PLK1 alone (lanes 1–3) or in the presence of purified Ku heterodimer (lanes 4–6). Where indicated, reactions contained the DNA-PK inhibitor NU7441 (lanes 2 and 6) or the PLK1 inhibitor BI2536 (lane 3) as indicated. All lanes contained DNA. ATP was present in lanes 1–3 and 5 and 6 as indicated. Reactions were stopped by boiling the samples in SDS sample buffer, and samples were run on an SDS 8%PAGE gel and probed with the described antibodies.

We previously identified Ser^3205^ as an *in vitro* DNA-PKcs autophosphorylation site [9] and showed that Ser^3205^ is phosphorylated in an ATM-dependent manner after IR [15]. Several high-throughput mass spectrometry screens have reported that Ser^3205^ is highly phosphorylated in mitosis [25,26,36,37]. Notably, Olsen et al. showed that the stoichiometry of phosphorylation of DNA-PKcs at Ser^3205^ was 14% in asynchronously growing HeLa cells, over 82% at mitosis and less than 10% in G1 and S, suggesting that Ser^3205^ phosphorylation is dynamically regulated during mitosis [36]. To confirm mitotic phosphorylation of DNA-PKcs Ser^3205^ and to identify the protein kinase responsible for its phosphorylation, DNA-PKcs was immunoprecipitated from cells that were treated with 40 ng/ml nocodazole for 15 h, then either untreated, or preincubated with the DNA-PKcs inhibitor (NU7441) or the ATM inhibitor (KU55933) for a further 1 h and isolated by mitotic shake off. Although, NU7441 blocked mitotic phosphorylation of DNA-PKcs at Ser^2056^ (Figure 3A), confirming autophosphorylation of this site in mitosis, neither NU7441 nor KU55933 affected phosphorylation at Ser^3205^ indicating that neither DNA-PK nor ATM is required for Ser^3205^ phosphorylation in mitosis (Figure 3A).

We observed that Ser^3205^ partially conforms to a putative consensus sequence for PLK1 [38,39]. To determine whether PLK1 phosphorylates DNA-PKcs at Ser^3205^, mitotic cells were incubated with the PLK1 inhibitor BI2536 and DNA-PKcs was immunoprecipitated from mitotic extracts. Significantly, inhibition of PLK1 blocked phosphorylation of DNA-PKcs at Ser^3205^ in mitotic extracts, while inhibition of Aurora kinase A had no effect on DNA-PKcs Ser^3205^ phosphorylation, indicating that phosphorylation of DNA-PKcs on Ser^3205^ is PLK1 dependent in mitosis (Figure 3A). Inhibition of PLK1 blocked PLK1-Thr^210^ phosphorylation as expected, and also blocked Aurora A-Thr^288^ phosphorylation, consistent with PLK1 lying upstream of Aurora A late in mitosis [40].

To determine whether PLK1 phosphorylated DNA-PKcs on Ser^3205^ *in vitro*, purified PLK1 (a kind gift from Dr James Hastie and Dr Dario Alessi, University of Dundee, Scotland, U.K.) was incubated with purified DNA-PK, dsDNA and either inhibitors to DNA-PKcs (NU7441) or PLK1 (BI2356) as indicated (Figure 3B). As expected, purified DNA-PKcs underwent autophosphorylation on Ser^3205^; however, PLK1 was also able to phosphorylate DNA-PKcs on Ser^3205^ *in vitro* (Figure 3B), suggesting that PLK1 directly targets DNA-PKcs on Ser^3205^ in mitosis.

### DNA-PKcs phosphorylated at Ser^3205^ localizes to the midbody at cytokinesis

**Figure 4:**
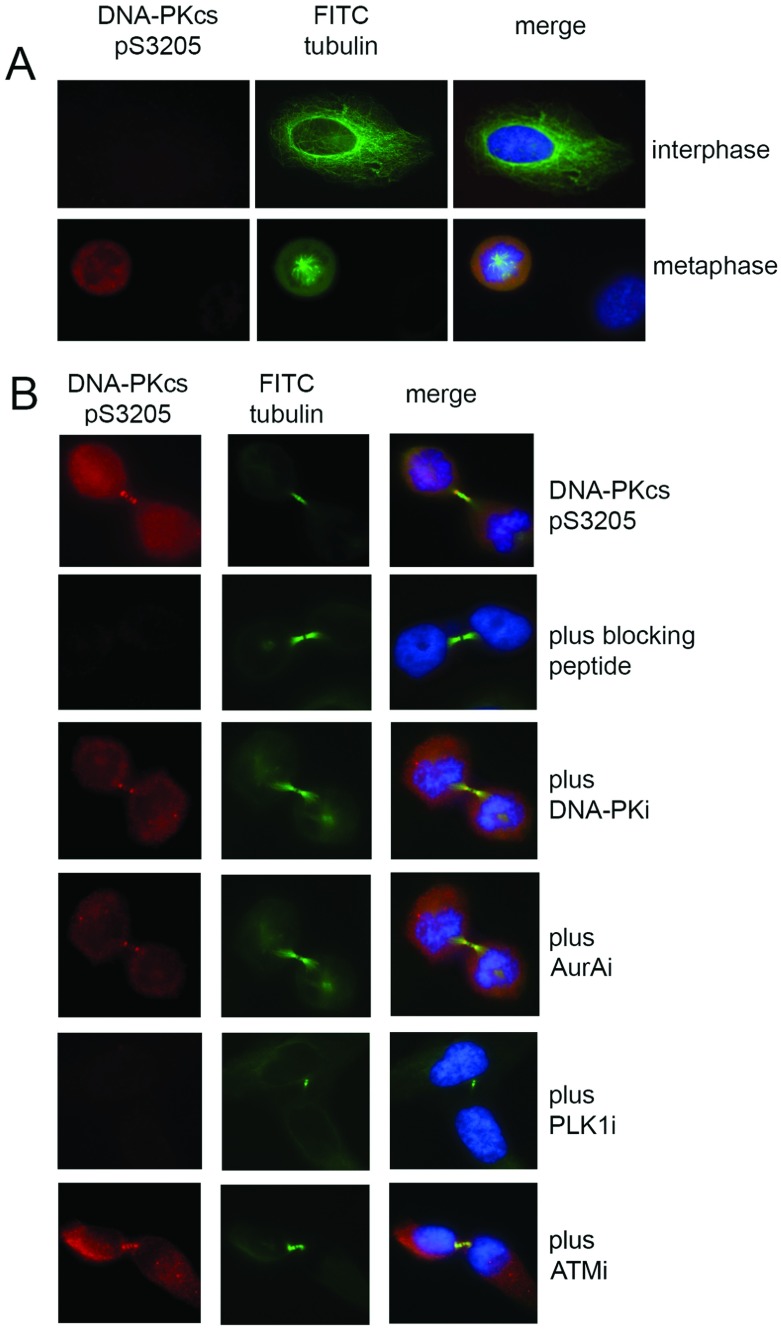
DNA-PKcs phosphorylated on Ser^3205^ localizes to the midbody in mitosis (**A**) U2OS cells were stained with FITC-conjugated α-tubulin (green) (1:1000 dilution), and a phosphospecific antibody to DNA-PKcs phospho-Ser^3205^ (red) (1:200 dilution). DNA was stained with DAPI (blue). (**B**) U2OS cells were treated with 100 nM of the PLK1 inhibitor (BI2536), 100 nM of Aurora-A-Inhibitor-I, 8 μM of the DNA-PK inhibitor (NU7441), or 5 μM of the ATM inhibitor (KU55933) for one hour prior to fixation and stained as above. Also shown as a control experiment (panel 2) in which U2OS cells were stained with FITC-conjugated α-tubulin (green) and the phosphospecific antibody to DNA-PKcs phosphoserine-3205 (red) in the presence of the 10 μg/ml phospho-blocking peptide. DNA stained with DAPI is shown in blue. Additional controls for the specificity of the phospho-Ser^3205^ phosphospecific antibody are shown in Supplementary Figure S3(C).

Phosphorylation of DNA-PKcs at Ser^3205^ in mitosis was further confirmed by immunofluorescence in U2OS cells. Ser^3205^ phosphorylation was not detected in interphase cells but was clearly present in mitotic cells (Figure 4A) and at the midbody in cytokinesis (Figure 4B). DNA-PKcs Ser^3205^ phosphorylation was resistant to inhibition of DNA-PK, ATM or Aurora A but was ablated by inhibition of PLK1 (Figure 4B). Together, these studies identify DNA-PKcs Ser^3205^ as a new target for PLK1 in mitosis and show that, like PLK1, DNA-PKcs phosphosphorylated on Ser^3205^ localizes to the midbody.

### The DNA-PKcs–PLK1 interaction is not mediated by phosphorylation

**Figure 5:**
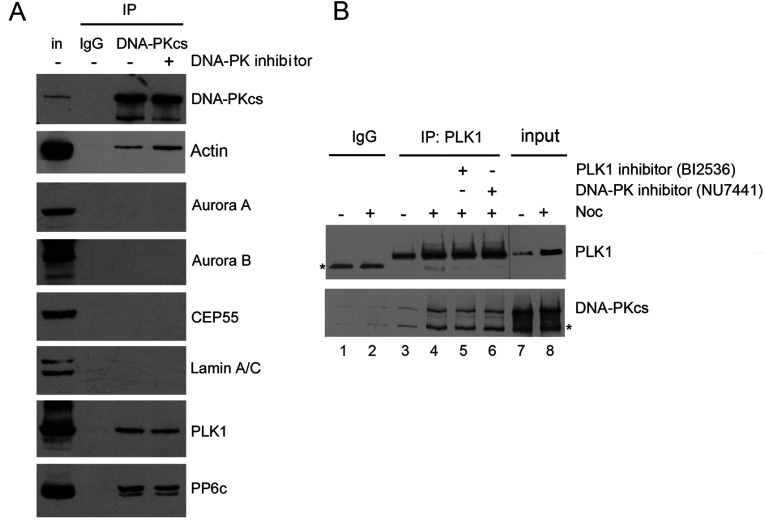
PLK1 interacts with DNA-PKcs in mitotic cells (**A**) DNA-PK immunoprecipitations were carried out from extracts of mitotic cells as in Figure 3. Samples were boiled in SDS–PAGE sample buffer, run on SDS-PAGE gels and probed using the antibodies indicated. (**B**) PLK1 immunoprecipitations were carried out from asynchronously growing or nocodazole treated cells as described in the Materials and Methods section. Where indicated, cells were treated with the DNA-PK inhibitor (8 μM) or the PLK1 inhibitor (100 nM) for 1 h prior to harvest. The asterisk represents a breakdown product of DNA-PKcs.

It was recently reported that PLK1, which contains an N-terminal FHA (forkhead associated) domain, interacts with DNA-PKcs in mitosis [41]. Given that FHA domains frequently mediate phospho-protein interactions [42], we asked whether the interaction between PLK1 and DNA-PKcs was mediated by phosphorylation. Mitotic cells were harvested after nocodazole treatment and shake off then incubated for 1 h with either NU7441 to inhibit DNA-PKcs or BI2536 to inhibit PLK1. DNA-PKcs was then immunoprecipitated and probed for the presence of DNA-PKcs and PLK1. The PLK–DNA-PKcs interaction was not disrupted by the addition of DNA-PK or PLK1 inhibitors, suggesting that it is not mediated by phosphorylation-dependent interactions (Figure 5 and Supplementary Figure S4A at http://www.bioscirep.org/bsr/034/bsr034e113add.htm). The DNA-PKcs–PLK1 interaction was also not disrupted by treatment with the broad-spectrum nuclease, benzonase (Supplementary Figure S4B), indicating that it is not DNA or RNA mediated. DNA-PKcs immunoprecipitates were also probed for PP6 and known mitotic proteins. Our results also show that DNA-PKcs interacts with PP6 and actin in mitotic extracts but not with Aurora A, Aurora B, CEP55 or nuclear lamins (Figure 5A).

### PP6 dephosphorylates DNA-PKcs Ser^3205^ in mitosis and after DNA damage

**Figure 6:**
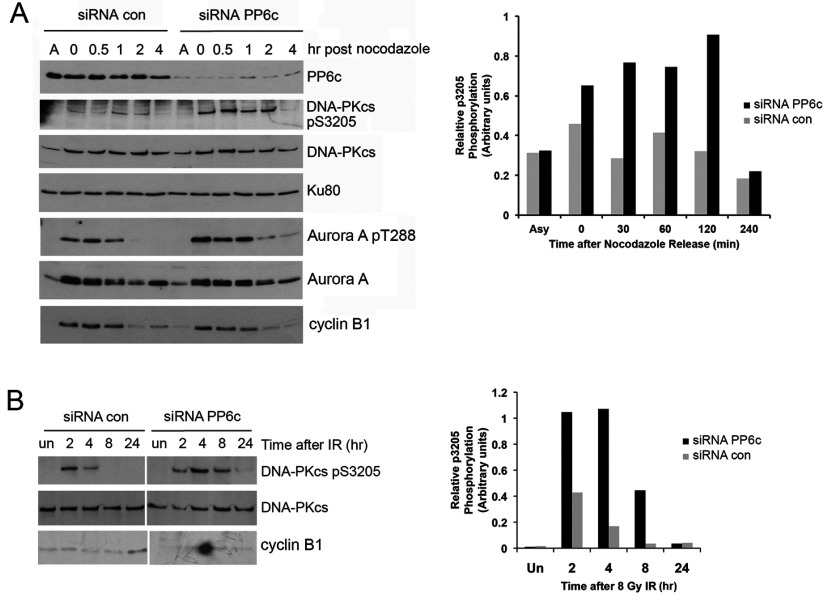
PP6 dephosphorylates DNA-PKcs phospho-Ser^3205^ in mitosis and after IR (**A**) HeLa cells were treated with siRNA to PP6c or scrambled control for 80 h and either left untreated (Asy) or treated with 40 ng/ml nocodazole for a further 16 h. Cells were harvested by mitotic shake off and left to recover in fresh media (minus nocodazole) for the times indicated. NETN lysates were prepared and DNA-PKcs immunoprecipitations were carried out for 2 h as described in the Materials and Methods section. Samples were boiled in SDS–PAGE sample buffer, run on an SDS/8%PAGE gel and probed for DNA-PKcs, DNA-PKcs-phospho-Ser^3205^, Ku80 and Aurora A as indicated. A graph showing the quantitation of Ser^3205^ phosphorylation normalized to total DNA-PKcs is shown on the right. These results are representative of three separate experiments. Immunoblots were also probed for cyclin B1 to confirm arrest in, and subsequent release from, mitosis. (**B**) HeLa cells were treated with siRNA to PP6c or scrambled control. Ninety-six hours after transfection, cells were either left untreated or treated with 8 Gy IR then left to recover for the times indicated. DNA-PK immunoprecipitations were carried out as described in the Materials and Methods section. Samples were boiled in SDS–PAGE sample buffer, run on an SDS/8%PAGE gel and probed using the antibodies described. All lanes are from the same exposure of the same gel but the image was cut to make the figure comparable to panel A. A graph showing the quantitation of Ser^3205^ phosphorylation normalized to total DNA-PKcs protein is shown on the right. The results are representative of three separate experiments. Blots were also probed for cyclin B1 expression under identical conditions to those in panel A. The absence of cyclin B1 staining indicates that PP6c depletion did not induce mitotic arrest.

DNA-PKcs interacts with PP6 [19,20] and PP6 dephosphorylates mitotic protein kinase Aurora A at Thr^288^, and has a major role in mitosis [21]. We therefore asked whether PP6 dephosphorylates Ser^3205^ of DNA-PKcs in mitosis. HeLa cells were transfected with either scrambled control siRNA or siRNA to PP6c, then incubated with nocodazole (40 ng/ml, 16 h) and either harvested immediately (time 0), or placed in fresh media (minus nocodazole) and allowed to recover for up to 4 h as indicated. Arrest and subsequent release from mitosis in nocodazole treated cells was confirmed by probing immunoblots for cyclin B1, which is expressed in early mitosis but degraded for cells to exit mitosis [43]. As reported previously, siRNA depletion of PP6 resulted in enhanced phosphorylation of Aurora A on Thr^288^ [21] (Figure 6A and Supplementary Figure S5 at http://www.bioscirep.org/bsr/034/bsr034e113add.htm). Significantly, siRNA depletion of PP6c enhanced the phosphorylation of DNA-PKcs at Ser^3205^ in mitosis (Figure 6A) and after IR (Figure 6B), revealing Ser^3205^ of DNA-PKcs as a new substrate of PP6 in mitosis and after DNA damage.

### PLK1 is not required for DNA-PK-dependent phosphorylation of Chk2 Thr^68^ in mitosis

**Figure 7:**
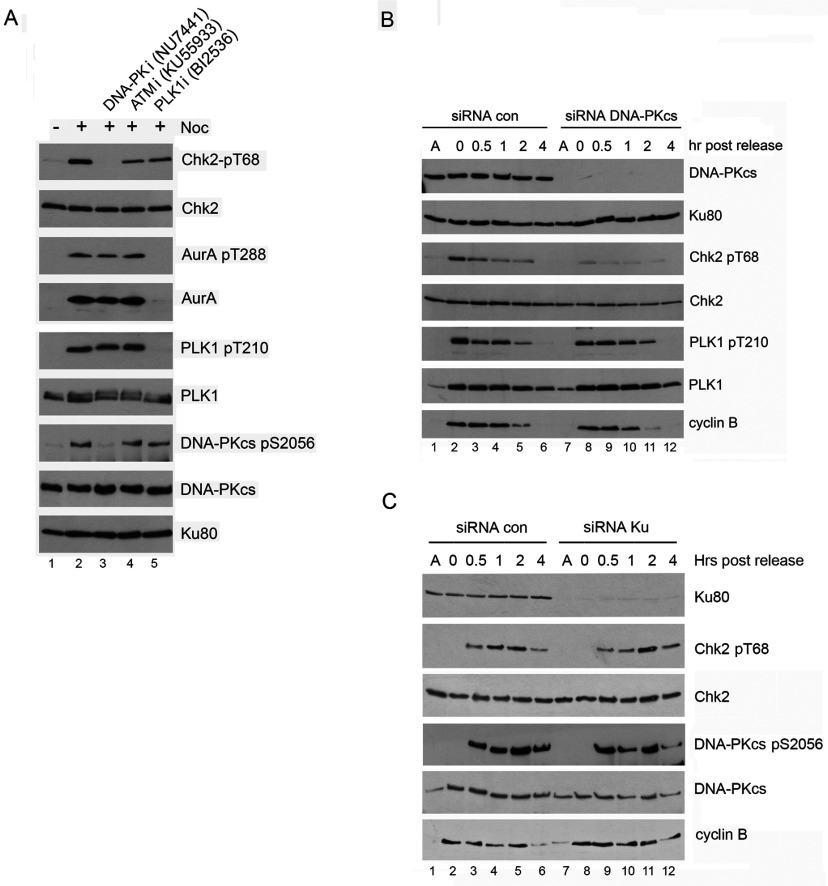
Inhibition of PLK1 does not affect DNA-PK dependent, mitotic phosphorylation of Chk2 on Thr^68^ (**A**) HeLa cells were left untreated or were treated with 40 ng/ml nocodazole for 16 h. One hour prior to mitotic shake off and harvesting, cells were left untreated or were treated with the DNA-PK inhibitor NU7441 (8 μM), the ATM inhibitor KU55933 (5 μM) or the PLK1 inhibitor BI2536 (100 nM). After mitotic shake-off the cells were left to recover for 35 min in the presence of the kinase inhibitors but in the absence of nocodazole. NETN lysates were prepared as described in the Materials and Methods section and 50 μg of extract was resolved on SDS–PAGE gels and probed with the antibodies as described. As in Figure 3(A), inhibition of PLK1 with BI2536 resulted in loss of phosphorylation of both PLK1 and Aurora A. (**B**) HeLa cells were transfected with siRNA to DNA-PKcs as described in the Materials and Methods section. At 80 h post-transfection, 40 ng/ml nocodazole was added for a further 16 h as indicated or cells were left untreated. Cells were harvested by mitotic shake-off and left to recover in nocodazole-free media for the times indicated. NETN lysates were prepared as described in the Materials and Methods section and 50 μg of extract was resolved on SDS–PAGE gels and probed with the antibodies as described. Quantitation of Figure 7(B) is shown in Supplementary Figure S6. Results are representative of three separate experiments. (**C**) HeLa cells were transfected with siRNA to siRNA to Ku70/80 and analyzed as described in Panel B. Quantitation of Figure 7(C) is shown in Supplementary Figure S6. Results are representative of three separate experiments.

After DNA damage, the cell-cycle checkpoint protein kinase Chk2 is phosphorylated on Thr^68^ in an ATM-dependent manner [44]. ATM-dependent activation of Chk2 leads to activation of cell-cycle checkpoint arrest at G1/S and G2/M [42,45]. However, recent studies also suggest a role for Chk2 in mitosis. Chk2 phosphorylates BRCA1 in mitosis in the absence of DNA damage [30,46] and co-localizes with PLK1 at the midbody [47]. In addition, lagging chromosomes, as observed here in DNA-PKcs depleted cells (Supplementary Figure S2C), are also a feature of Chk2-deficient cells [30]. Moreover, Chk2 phosphorylation on Thr^68^ and BRCA1 phosphorylation on Ser^988^ in mitosis requires the activity of DNA-PK [28,29], suggesting that DNA-PKcs regulates both Chk2 and BRCA1 in mitosis to regulate genome stability. Given that PLK1 phosphorylates DNA-PKcs on Ser^3205^ in mitosis, we asked whether inhibition of PLK1 affected DNA-PKcs mediated phosphorylation of Chk2 in mitosis. HeLa cells were incubated with nocodazole for 15 h, followed by incubation with the DNA-PK inhibitor NU7441, the ATM inhibitor KU55933 or the PLK1 inhibitor BI2536 for 1 h. After mitotic shake off, cells were examined for Chk2 Thr^68^ phosphorylation. Mitotic phosphorylation of Chk2 at Thr^68^ was abrogated by incubation with the DNA-PK inhibitor NU7441 confirming recent studies indicating that DNA-PK phosphorylates Chk2 on Thr^68^ in mitosis [28,29], however, phosphorylation was unaffected by incubation with the ATM inhibitor KU55933 or the PLK1 inhibitor BI2536 (Figure 7A). Thus, as demonstrated in Figure 3(A), PLK1-dependent phosphorylation of DNA-PKcs is not required for DNA-PK-dependent activation of Chk2 in mitosis.

### Autophosphorylation of DNA-PKcs and DNA-PKcs-dependent phosphorylation of Chk2 at Thr^68^ in mitosis is independent of Ku and DNA damage

**Figure 8:**
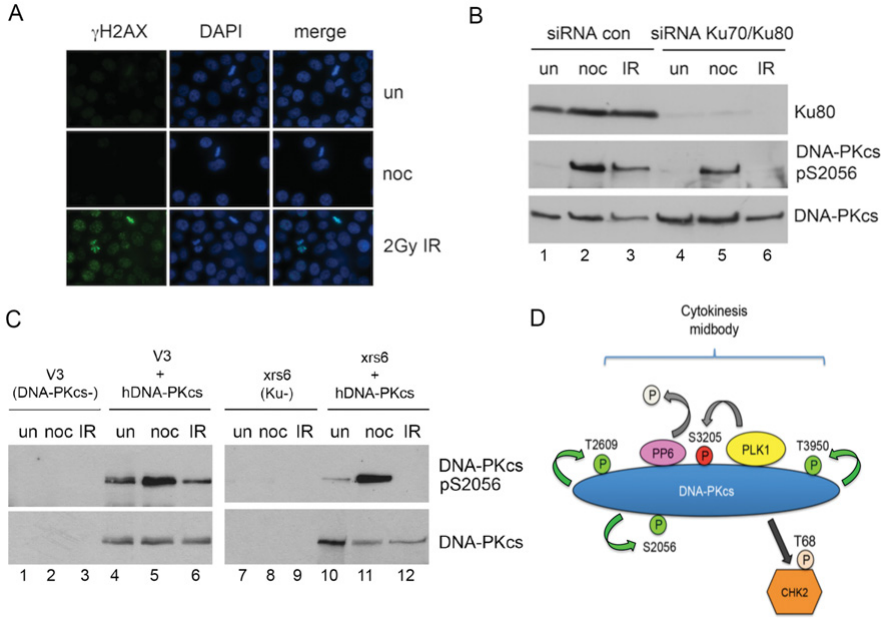
DNA-PKcs is activated in mitosis in the absence of Ku and DNA damage (**A**) HeLa cells were grown on poly-L-lysine coated coverslips and either left untreated, treated with 40 ng/ml nocodazole for 16 h or treated with 2 Gy IR and allowed to recover for 1 h. Cells were fixed and stained for γ-H2AX as described previously [19]. (**B**) HeLa cells were transfected with scrambled siRNA control or siRNA to Ku (100 nM Ku70 siRNA, 100 nM Ku80 siRNA) as described in Figure 7(B). Eighty hours post transfection, cells were untreated, or treated with 40 ng/ml nocodazole for 16 h. One hour prior to harvesting, where indicated, cells were treated with 10 Gy IR and left to recover. Nocodazole-treated cells were harvested by mitotic shake-off as described, and untreated and IR treated cells were harvested by trypsinization. NETN lysates were prepared as described in the Materials and Methods section and 50 μg of extracts were resolved on an 8% SDS–PAGE gel and probed for the antibodies indicated. (**C**) DNA-PK null V3 rodent cell (lanes 1–3), V3 cells stably expressing GFP-tagged human DNA-PKcs (lanes 4–6), Ku null xrs-6 rodent cells (lanes 7–9) or Ku null xrs-6 rodent cells stably expressing GFP-tagged human DNA-PKcs (lanes 10–12) were either untreated, treated with 40 ng/ml nocodazole for 16 h or irradiated 10 Gy, 1 h recovery as above. NETN lysates were prepared and immunoprecipitated with antibodies to GFP as described in Material and Methods. Samples were run on 8% SDS–PAGE gels and probed for the antibodies indicated. (**D**) Model for role of DNA-PKcs in mitosis. DNA-PKcs interacts with PP6 and PLK1 in mitosis. DNA-PKcs undergoes autophosphorylation at Thr^2609^, Thr^3950^ and Ser^2056^ in mitosis (indicated by green arrows and green circles). Ser^3205^ is phosphorylated by PLK1 (indicated by the red circle) and dephosphorylated by PP6 in mitosis. DNA-PKcs phosphorylated at Thr^2609^, Ser^3205^ and Thr^3950^ localizes to the midbody in cytokinesis. Also shown is DNA-PKcs dependent phosphorylation of Chk2 on Thr^68^.

Phosphorylation of Chk2 on Thr^68^ after DNA damage requires ATM [48], whereas Thr^68^ phosphorylation of Chk2 in nocodazole-treated cells requires DNA-PK ([28,29] and Figure 7A). It is well established that in response to DNA damage, the Ku protein binds DSBs, which in turn recruits DNA-PKcs, stimulating its protein kinase activity [49,50]. We therefore asked whether the nocodazole conditions used in our experiments induced DNA damage, which would be predicted to induce Ku-dependent activation of DNA-PKcs. HeLa cells were treated with nocodazole at 40 ng/ml for 16 h and assayed for γ-H2AX phosphorylation, a well-established marker of DSBs [51]. No γ-H2AX foci were detected in nocodazole treated cells (Figure 8A), indicating that the nocodazole conditions used in our experiments do not induced DSBs.

We next examined whether activation of DNA-PKcs in mitosis required Ku. Ku70 and Ku80 were depleted by siRNA then cells were either untreated, irradiated (10 Gy IR) and harvested after 1 h, or treated with nocodazole as above. Immunoblots were then probed for DNA-PKcs phosphorylation on Ser^2056^, an established DNA damage induced autophosphorylation site [52,53]. Although Ku was depleted by over 95%, nocodazole induced phosphorylation of DNA-PKcs on Ser^2056^ was only slightly reduced, whereas IR-induced phosphorylation of DNA-PKcs on Ser^2056^ was virtually eliminated (Figure 8B). To further investigate this apparent lack of requirement for Ku, Ku-deficient rodent xrs6 cells stably expressing human DNA-PKcs were either treated with nocodazole or IR. Again, loss of Ku abrogated the IR-induced phosphorylation of DNA-PKcs at Ser^2056^, compared with Ku-proficient, DNA-PKcs-deficient V3 rodent cells expressing human DNA-PKcs, while nocodazole-induced phosphorylation of DNA-PKcs at Ser^2056^ was relatively unaffected (Figure 8C). These results suggest that the nocodazole conditions used here do not induce DNA damage and that Ku is not required for activation of DNA-PKcs in mitosis.

To determine whether Chk2 Thr^68^ phosphorylation was also Ku independent, DNA-PKcs or Ku were depleted from HeLa cells using siRNA, then cells were treated with nocodazole, harvested by shake off and either analysed immediately or after incubation in nocodazole-free media for 0.5, 1, 2 or 4 h. Depletion of DNA-PKcs reduced Chk2 Thr^68^ phosphorylation, whereas depletion of Ku70/80 had relatively little effect on Chk2 Thr^68^ phosphorylation (Figures 7B and 7C and Supplementary Figure S6 at http://www.bioscirep.org/bsr/034/bsr034e113add.htm). Together, these results suggest that neither autophosphorylation of DNA-PKcs nor DNA-PKcs-dependent phosphorylation of Chk2 in mitosis requires Ku and that both occur in the absence of DNA damage. Thus, activation of DNA-PKcs in mitosis appears to be mechanistically different from Ku-dependent activation of DNA-PKcs after DNA damage.

## DISCUSSION

Previous studies have shown that DNA-PKcs and PP6 are required for faithful mitosis and that DNA-PKcs is phosphorylated on Ser^2056^, Thr^2609^ and Thr^2647^ in mitosis [21,23,27]. Mitotic phosphorylation at these sites was DNA-PK-dependent and DNA-PKcs phosphorylated at Thr^2609^ and Thr^2647^ localized to the midbody in cytokinesis [23,27]. Here, we show that Thr^3950^, which is located in the putative activation loop, in the catalytic domain of DNA-PKcs [18], is also autophosphorylated in mitosis. Like DNA-PKcs phosphorylated on Thr^2609^ and Thr^2647^, DNA-PKcs phosphorylated at Thr^3950^ localizes to centrosomes in metaphase and the midbody in cytokinesis.

We also show that Ser^3205^, which is located in the conserved FAT domain of DNA-PKcs (Figure 1A), is phosphorylated in mitosis in a PLK1-dependent manner *in vivo*. Moreover, PLK1 phosphorylates DNA-PKcs directly on Ser^3205^ *in vitro*, suggesting that DNA-PKcs Ser^3205^ is a direct target of PLK1 in mitosis. We also confirm that PLK1 physically interacts with DNA-PKcs in mitosis and show that this interaction is resistant to inhibition of either DNA-PKcs or PLK1 therefore is not mediated by phosphorylation of either protein. Our results confirm that DNA-PKcs phosphorylates Chk2 on Thr^68^ in mitosis [29] and reveal that, despite being required for Ser^3205^ phosphorylation in mitosis, PLK1 is not required for Chk2 Thr^68^ phosphorylation, and, by implication, activation, of Chk2 in mitosis.

Thr^3950^-phosphorylated DNA-PKcs was absent in interphase cells in the absence of DNA damage but was detected at the centrosomes during prophase and metaphase (Figure 2). Like phosphorylation at Thr^2609^, DNA-PKcs phosphorylation at Thr^3950^ was detected at the midbody. (Figure 2 and Supplementary Figure S3A), as was Thr^210^ phosphorylated PLK1 (Supplementary Figure S3B). Ser^3205^ phosphorylation was also not detected in interphase cells in the absence of DNA damage but, unlike phospho-Thr^3950^, phospho-Ser^3205^ had a diffuse staining pattern in metaphase cells and was present at the midbody in cytokinesis, suggesting different modes of regulation of DNA-PKcs at phospho-Thr^3950^ and phospho-Ser^3205^ during mitosis.

Importantly, our studies raise several important questions regarding how DNA-PKcs is activated in mitosis. The nocodazole treatment used here did not induce γ-H2AX phosphorylation, and phosphorylation of Chk2 in nocodazole-treated cells was DNA-PK-dependent, rather than ATM-dependent as it is after DNA damage [48]. Moreover, our results from siRNA experiments in human cells and in Ku defective *xrs-6* rodent cells are all consistent with both mitotic autophosphorylation of DNA-PKcs and mitotic phosphorylation of Chk2 being Ku-independent. Thus, our results indicate that activation of DNA-PKcs in mitosis occurs in the absence of DNA damage and is mechanistically distinct from the manner in which it is activated following DNA damage. We have previously shown that DNA-PKcs can be activated by tethering of its amino terminus [32], thus it is possible that conformational changes induced by post-translational modification or interaction with other mitotic proteins could lead to activation of DNA-PKcs in the absence of Ku and DNA damage.

Our studies also provide biochemical evidence for association of PP6 catalytic and regulatory subunits with mitotic spindles. Interestingly, the presence of protein phosphatase inhibitors reduced the association of PP6 subunits with spindles while enhancing Aurora A Thr^288^ phosphorylation. This is in agreement with the results of Barr and colleagues, who demonstrated that Aurora A protein kinase activity is regulated by PP6 during mitosis [21]. Our results suggest that Aurora A phosphorylation may be directly/indirectly regulated by the tightly controlled interaction of PP6 with mitotic spindles. Previous studies have identified γ-H2AX [19] and Aurora A [21] as targets of PP6 but, despite the fact that PP6 interacts with DNA-PKcs, previous experiments failed to show that PP6 was able to dephosphorylate DNA-PKcs at autophosphorylation sites Ser^2056^ or Thr^2609^ [19,20]. Here, we show that PP6 is required for dephosphorylation of DNA-PKcs at the PLK1 phosphorylation site, Ser^3205^, in mitosis and after DNA damage.

Collectively our data provide mechanistic insight into the role of DNA-PKcs and PP6 in mitosis and suggest that PLK1 mediated phosphorylation of DNA-PKcs on Ser^3205^ is functionally distinct from DNA-PKcs-dependent phosphorylation of Chk2 on Thr^68^ in mitosis. Since inactivation of DNA-PKcs, PLK1, PP6 or Chk2 leads to mitotic defects including misaligned chromosomes, lagging chromosomes and abnormal nuclear morphologies and cytokinesis ([23,27,30,47] and Supplementary Figure S2), these findings have relevance to understanding the mechanism of aneuploidy in cancer cells.

## Online data

Supplementary data
