# Supplementary material for: Polo-like kinase 1 (PLK1) and protein phosphatase 6 (PP6) regulate DNA-dependent protein kinase catalytic subunit (DNA-PKcs) phosphorylation in mitosis
Source: Biosci Rep. 2014 Jun 25;34(3):e00113. doi: 10.1042/BSR20140051 (PMC4069685; doi:10.1042/BSR20140051)
Supplement: Supplementary data [file bsr034e113add.pdf]

Biosci. Rep. (2014) / **34** / art:e00113 / doi 10.1042/BSR20140051

OPEN ACCESS

Original Paper

Bioscience Reports

www.bioscirep.org

SUPPLEMENTARY DATA

Polo-like kinase 1 (PLK1) and protein phosphatase 6 (PP6) regulate DNA-dependent protein kinase catalytic subunit (DNA-PKcs) phosphorylation in mitosis

Pauline DOUGLAS*, Ruiqiong YE*, Laura TRINKLE-MULCAHY†, Jessica A. NEAL‡, Veerle De WEVER§[1], Nick A. MORRICE‖, Katheryn MEEK‡ and Susan P. LEES-MILLER*[2]

*Departments of Biochemistry & Molecular Biology and Oncology, Southern Alberta Cancer Research Institute, University of Calgary, 3330 Hospital Drive NW, Calgary, AB, Canada, T2N 4N1
†Department of Cellular & Molecular Medicine and Ottawa Institute of Systems Biology, University of Ottawa, Ottawa, Ontario, Canada, K1H 8M5
‡Departments of Pathobiology & Diagnostic Investigation, and Microbiology & Molecular Genetics, Michigan State University, East Lansing, Michigan, U.S.A.
§Department of Biological Sciences, University of Calgary, 2500 University Drive NW, Calgary, AB, Canada T2N 4N1
‖Beatson Institute for Cancer Research, Glasgow G61 1BD Scotland, U.K.

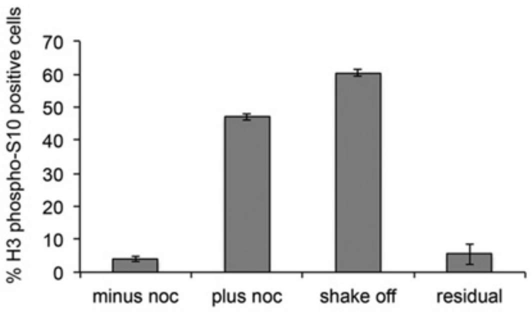

Figure S1 Nocodazole induces mitosis as indicated by histone H3 Ser^{10} phosphorylation
HeLa cells were either untreated or incubated with nocodazole (40 ng/ml) for 16 h then harvested by mitotic shake off. Aliquots of untreated cells, total nocodazole-treated cells (total), nocodazole-treated cells after shake off (shake off) and cells remaining after shake off (residual) were stained with FITC-conjugated histone H3-phospho-Ser^{10} antibody and analysed by flow cytometry as described previously [1]. Shown is the average of three separate experiments with standard deviation.

[1] Present address: Department of Cellular and Molecular Medicine, Laboratory of Biosignaling & Therapeutics, Campus Gasthuisberg, Leuven, Belgium
[2] To whom correspondence should be addressed (email leesmill@ucalgary.ca).

© 2014 The Author(s) This is an Open Access article distributed under the terms of the Creative Commons Attribution Licence (CC-BY) (http://creativecommons.org/licenses/by/3.0/) which permits unrestricted use, distribution and reproduction in any medium, provided the original work is properly cited.

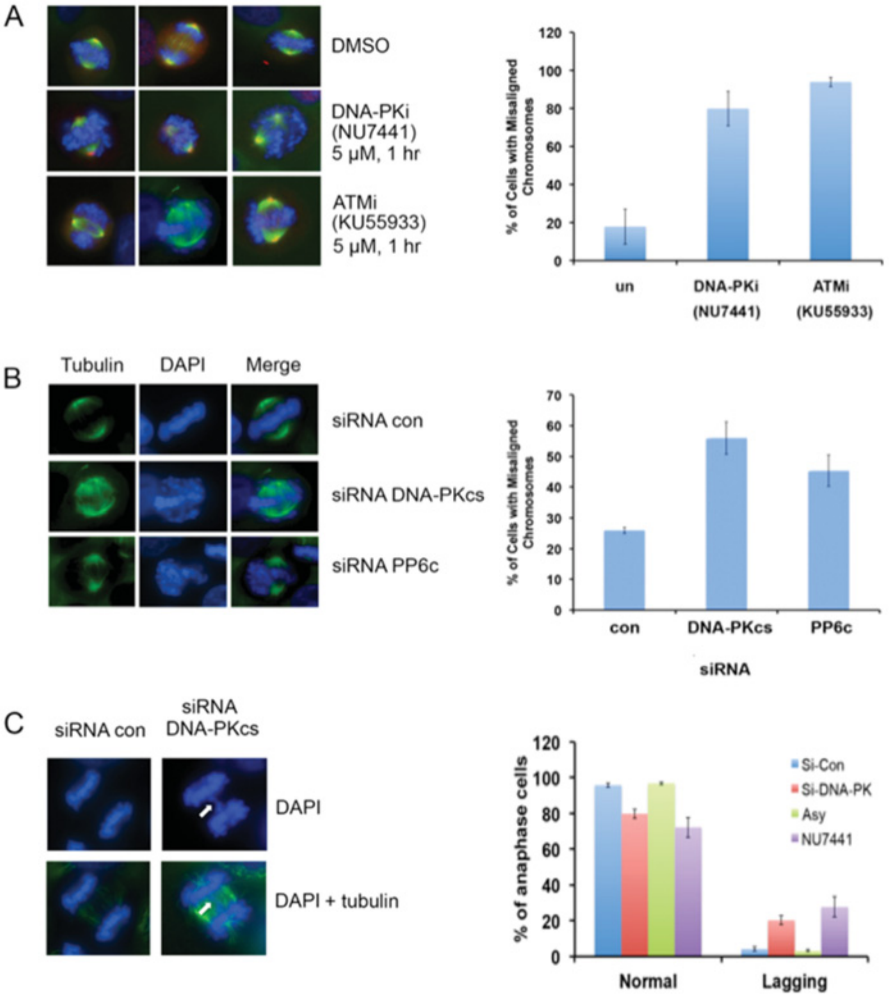

Figure S2 Depletion or inhibition of DNA-PK induces mitotic defects

(**A**) HeLa cells were grown on coverslips as described in the Materials and Methods section and treated with either DMSO, the DNA-PK inhibitor NU7441 (8 μM) or the ATM inhibitor KU55933 (5 μM) for 1 h prior to fixation with formaldehyde. Cells were processed for immunofluorescence as described in the Materials and Methods section. FITC-conjugated α-tubulin was used at 1:1000 dilution. The left panel shows representative images of normal and misaligned chromosomes. The graph on the right represents three experiments in which 500 mitotic cells were counted per experiment. The average with STD is shown. (**B**) siRNA to PP6c or DNA-PKcs was carried out in HeLa cells as described in the Materials and Methods section. Cells were processed for immunofluorescence as described above. The left panel shows representative images of normal and misaligned chromosomes. The graph on the right represents three experiments in which 500 mitotic cells were counted in each experiment. The average with STD is shown. (**C**) HeLa cells were treated with siRNA to DNA-PKcs as described, or were treated with the DNA-PK inhibitor NU7441 (8 μM) for 16 h. Cells were processed for immunofluorescence as described above. The left panel shows representative images of normal or lagging chromosomes (indicated by white arrows). The graph on the right represents three experiments in which 200 mitotic cells were counted per experiment. The average with STD is shown.

© 2014 The Author(s) This is an Open Access article distributed under the terms of the Creative Commons Attribution Licence (CC-BY) (http://creativecommons.org/licenses/by/3.0/) which permits unrestricted use, distribution and reproduction in any medium, provided the original work is properly cited.

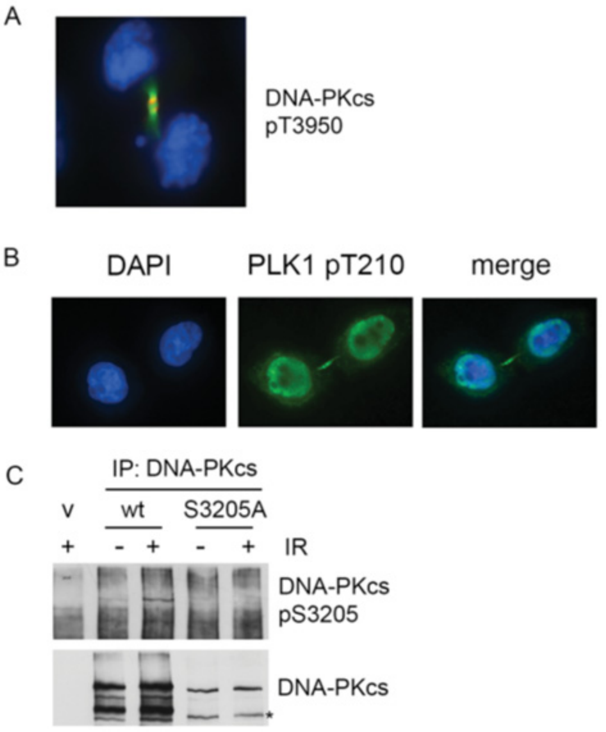

Figure S3 DNA-PKcs immunoprecipitates with PLK1
(**A**) U2OS cells processed for immunofluorescence as described in the Materials and Methods section. The DNA-PKcs phospho-Thr^{3950} antibody was used at 1:200 dilution. (**B**) U2OS cells were processed for immunofluorescence as described in the Materials and Methods section. The PLK1 phospho-Thr^{210} antibody was used at 1:500 dilution. (**C**) Control for DNA-PKcs phospho-Ser^{3205} antibody: DNA-PKcs was immunoprecipitated from V3 cells stably expressing wild-type DNA-PKcs or DNA-PKcs S3205A (described in [2]) as indicated. Where indicated, cells were irradiated (10 Gy 1 h). Immunoprecipitates were probed for DNA-PKcs and a phosphospecific antibody to Ser^{3205} as indicated. The * indicates a breakdown product of DNA-PKcs.

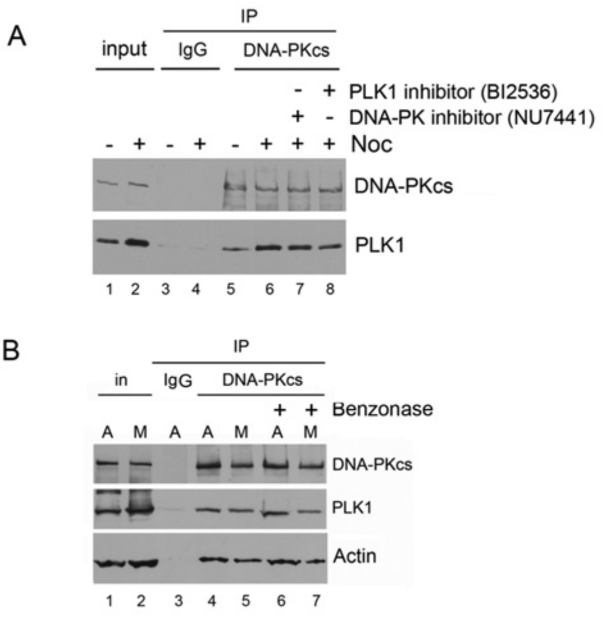

Figure S4 Thr^{3950}-phosphorylated DNA-PKcs localizes to the midbody
(**A**) DNA-PKcs was immunoprecipitated from nocodazole-treated cells as described in Figure 3. Where indicated, cells were pretreated for 1 h with the PLK1 inhibitor BI2356 (100 nM) or the DNA-PK inhibitor NU7441 (8 μM). Immunoprecipitates were boiled in SDS–PAGE sample buffer and run on a SDS/PAGE gel and probed for the antibodies indicated. (**B**) DNA-PK immunoprecipitations were carried out using 1 mg of NETN extract from either asynchronized [A] cells or mitotic shake-off cells [M] in the presence or absence of benzonase treatment as indicated. Immunoprecipitates were boiled in SDS–PAGE sample buffer and run on a SDS–PAGE gel and probed for the antibodies indicated.

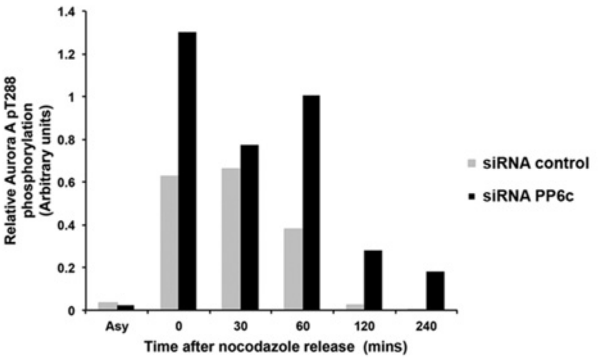

Figure S5 Effect of siRNA to PP6c and scrambled control on phosphorylation of Aurora A, Thr^{288}
Quantification of data shown in Figure 6(A) of the main text.

© 2014 The Author(s) This is an Open Access article distributed under the terms of the Creative Commons Attribution Licence (CC-BY) (http://creativecommons.org/licenses/by/3.0/) which permits unrestricted use, distribution and reproduction in any medium, provided the original work is properly cited.

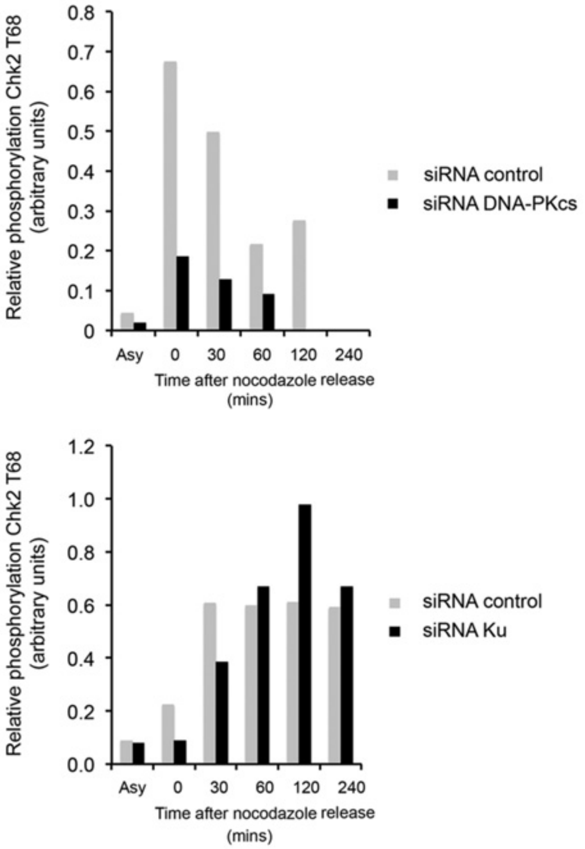

Figure S6 Effects of siRNA depletion of DNA-PKcs and Ku on Chk2 Thr^{68} phosphorylation in mitosis
Quantification of data shown in Figures 7(B) and 7(C) of the main text. The results are representative of at least three separate experiments.

REFERENCES

1 Douglas, P., Zhong, J., Ye, R., Moorhead, G. B., Xu, X. and Lees-Miller, S. P. (2010) Protein phosphatase 6 interacts with the DNA-dependent protein kinase catalytic subunit and dephosphorylates gamma-H2AX. Mol. Cell. Biol. **30**, 1368–1381 CrossRef PubMed

2 Neal, J. A., Dang, V., Douglas, P., Wold, M. S., Lees-Miller, S. P. and Meek, K. (2011) Inhibition of homologous recombination by DNA-dependent protein kinase requires kinase activity, is titratable, and is modulated by autophosphorylation. Mol. Cell. Biol. **31**, 1719–1733 CrossRef PubMed

Received 1 April 2014/25 April 2014; accepted 20 May 2014

Published as Immediate Publication 20 May 2014, doi 10.1042/BSR20140051

© 2014 The Author(s) This is an Open Access article distributed under the terms of the Creative Commons Attribution Licence (CC-BY) (http://creativecommons.org/licenses/by/3.0/) which permits unrestricted use, distribution and reproduction in any medium, provided the original work is properly cited.
